# Subcutaneous Vulvar Flap Viability Evaluation With Near-Infrared Probe and Indocyanine Green for Vulvar Cancer Reconstructive Surgery: A Feasible Technique

**DOI:** 10.3389/fsurg.2021.721770

**Published:** 2021-08-09

**Authors:** Vito Andrea Capozzi, Luciano Monfardini, Giulio Sozzi, Giulia Armano, Andrea Rosati, Salvatore Gueli Alletti, Francesco Cosentino, Alfredo Ercoli, Stefano Cianci, Roberto Berretta

**Affiliations:** ^1^Department of Medicine and Surgery, University of Parma, Parma, Italy; ^2^Department of Gynecologic Oncology, University of Palermo, Palermo, Italy; ^3^Department of Women's and Children's Health, Fondazione Policlinico Universitario A. Gemelli IRCCS, Rome, Italy; ^4^Department of Medicine and Health Science “V.Tiberio”, Università degli Studi del Molise, Campobasso, Italy; ^5^Department of Human Pathology of the Adult and of the Childhood “Gaetano Barresi”, Università di Messina, Messina, Italy

**Keywords:** vulvar cancer, indocyanine green, laparoscopic near-infrared probe, flap viability, vulvar flap

## Abstract

**Introduction:** Vulvar cancer is a rare condition affecting older women and accounts for 3–5% of all gynecological cancers. Primary surgical treatment involves the removal of a large amount of tissue for which reconstructive surgery is often necessary with a high rate of postoperative complications. Despite several techniques for the evaluation of vulvar flap viability have been proposed, many methods cannot be performed during surgery and require expensive devices often missing in a gynecological clinic. This study aims to verify the feasibility and the safety of the vulvar flap viability evaluation through a near-infrared endoscopic probe and Indocyanine green (ICG) tracer in a small group of patients and to evaluate long-term vulvar flap outcomes.

**Methods:** Patients with primary vulvar cancer who required surgical treatment and subsequent vulvar flap reconstructive surgery were prospectively included in the study. A 25 mg ICG vial diluted in 20 ml of saline solution was intravenously infused before closing the skin edges of the flaps. All patients were given 0.2 mg/kg body weight of intravenous ICG. After 10–15 min, a near-infrared endoscopic probe was used to evaluate the vulvar flap viability.

**Results:** Of the 18 patients who underwent radical vulvectomy for vulvar cancer during the study period, 15 were included in the analysis. All packaged surgical flaps showed tracer uptake on the surgical margin. No intro-operative complications were recorded neither surgery-related nor to dye infusion. No surgical infection, dehiscence, or necrosis was recorded.

**Conclusions:** Vulvar flap viability assessment using Indocyanine green and a laparoscopic infrared probe is a feasible method. All cases included in the analysis showed a dye uptake on the surgical edge of the flap. Further, prospective studies are needed to confirm the method in clinical practice and to evaluate its superiority over simple subjective clinical evaluation.

## Introduction

Vulvar cancer is a rare condition affecting older women and accounts for 3–5% of all gynecological cancers (1). Primary surgical treatment is radical vulvectomy with inguinal nodal staging (2). A disease-free margin of 8 mm by the primary lesion should be ensured, and this could lead to extensive demolition surgery which may require a plastic reconstructive surgical time (3).

Reconstructive surgery should replace the removed tissue with a healthy donor area through tension-free skin closure. Although, multiple reconstructive surgical techniques have been proposed over the years, the rate of postoperative complications remains high (4, 5). In addition, surgical complications for malignant gynecological pathology are affected by a high complication rate (6). In particular, wound dehiscences, low genital tract infections, vaginal and anovaginal shrinkage, urethral damage, and anti-aesthetic reconstructions may worsen the patient's quality of life and could require reintervention (7–11).

The fasciocutaneous advancement flaps are the most used by the oncologist gynecologist for their easier feasibility with satisfactory surgical outcomes (12). Hand et al. reported 33 and 15% of surgical dehiscence and infectious complications, respectively with V-Y fasciocutaneous flap reconstruction (13).

A key point for the vulvar flap success is the evaluation of the recovered tissue viability after its displacement. Despite several techniques for the evaluation of vulvar flap viability have been proposed, many methods cannot be performed during surgery and require expensive devices often missing in a gynecological clinic (14–16). Angiography with Indocyanine green (ICG) shows a 90% sensitivity in the flap vitality assessment but the clinical evaluation of color, turgor, and bleeding of the flap still represent a crucial aspect that should always be evaluated (17). Furthermore, a unanimous consensus on which is the best method to use has not been reached worldwide (2, 18).

We previously proposed for the first time the vulvar flap viability evaluation using a near-infrared endoscopic probe and ICG tracer (19). The method, applied on a single patient, proved to be safe, easy to perform and did not require missing devices in common gynecological clinics.

This study aims to verify the feasibility and the safety of the vulvar flap viability evaluation through a near-infrared endoscopic probe and ICG tracer in a small group of patients and to evaluate long-term vulvar flap outcomes.

## Methods

Patients with primary vulvar cancer who required surgical treatment and subsequent vulvar flap reconstructive surgery from January to November 2019 accessing the department of Gynecology and Obstetrics of the University of Parma were prospectively included in the pilot study.

According to the National Comprehensive Cancer Network (NCCN) guidelines, all patients underwent modulated surgery and subsequent adjuvant therapy related to the site and size of the primary lesion (2). The vulvar flap used was chosen concerning the site and the amount of tissue to be replaced (18).

A 25 mg ICG vial diluted in 20 ml of saline solution was intravenously infused before closing the skin edges of the flaps. After 10–15 min, a near-infrared endoscopic probe (Storz Endoscopic IMAGE 1SH3-link TC300 and Light source Storz D-light P 20133720) was used to evaluate the vulvar flap viability. A subsequent flap evaluation was performed after closing the skin edges of the flaps, 30 min after the ICG infusion. A double-check by two different operators to verify the flap viability was performed.

Anamnestic data, BMI, age at the time of surgery, type of surgery, type of reconstructive flap, International Federation of Gynecology and Obstetrics (FIGO) stage, intraoperative, and postoperative complications were collected for each patient.

All patients underwent clinical follow-up at our department every 3 months. All patients who did not perform reconstructive vulval flap surgery, patients who did not provide their informed consent, patients who did not perform a clinical follow-up for at least 6 months, or with known allergy to the contrast agents were excluded from the study. Furthermore, patients with multiple comorbidities, or with American Society of Anesthesiologists (ASA) status 3 were excluded from the analysis.

All surgical procedures, both in the demolition and reconstructive phase, were performed by gynecologists oncologists dedicated to vulvar cancer.

All patients provided their informed consent for the writing of the article and the publication of the images and videos in an anonymous format. The study was approved by the ethics committee of the University of Parma, with protocol number: 0001123.

## Results

Of the 18 patients who underwent radical vulvectomy for vulvar cancer in our department during the study period, 15 were included in the analysis. Two patients did not provide informed consent for study participation and one did not perform post-operative follow-up.

Median age was 74 years with standard deviation (sd) 9.6, median body mass index (BMI) was 29 (sd 4.2), 13 patients had squamous cancer, and two basaloid histology. Twelve patients were FIGO stage IB1, one IIIB, and two cases showed FIGO stage IIA. The median lesion diameter was 20 mm. All patients underwent radical vulvectomy, the sentinel lymph node was removed bilaterally in 3 patients, and bilateral inguinal lymphadenectomy was performed in two patients. All patients underwent reconstructive surgery after vulvectomy, 12 patients with a V-Y advancement flap, two with a lotus petal flap, and one with a combined V-Y flap and lotus petal flap for hemivulva. Patient characteristics are shown in Table 1. All packaged surgical flaps showed tracer uptake on the surgical margin. No intro-operative complications were recorded neither surgery-related nor to dye infusion. No surgical infection, dehiscence, or necrosis was recorded. One patient showed a lymphocele at the site of inguinal lymphadenectomy that was subsequently surgically drained.

**Table 1:** Patients' characteristics.

	**Number**	
Cases	15	
Age (median)	44 (sd 9.6)	
BMI (median)	29 (sd 4.2)	
Histological types		
	13	Squamous cell carcinoma
	2	Basaloid carcinoma
FIGO stage		
	12	IB1
	2	IIA
	1	IIIB
Demolition surgery		
	10	Radical vulvectomy
	3	Radical vulvectomy and inguinal sentinel lymph node
	2	Radical vulvectomy and inguinal lymphadenectomy
Reconstructive surgery		
	12	V-Y advancement flap
	2	Lotus petal flap
	1	Combined V-Y and lotus petal flap
Complication		
	0	Intraoperative
	1	Postoperative (Inguinal lymphocele)

*Sd, standard deviation; BMI, Body mass index; FIGO, International Federation of Gynecology and Obstetrics*.

**Figure 1:**
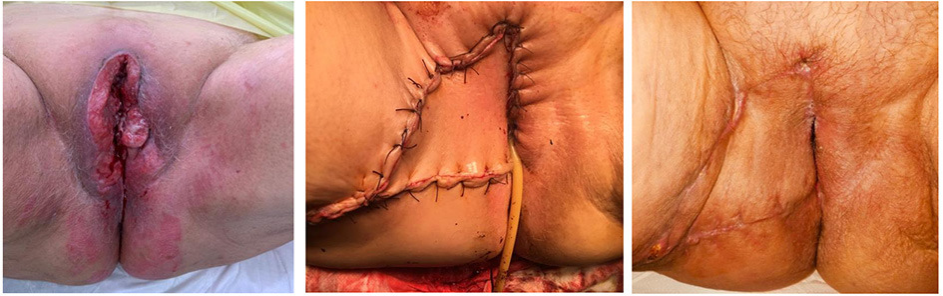
Fasciocutaneous advancement V-Y flap (preoperative, postoperative, and three-month follow-up).

**Figure 2:**
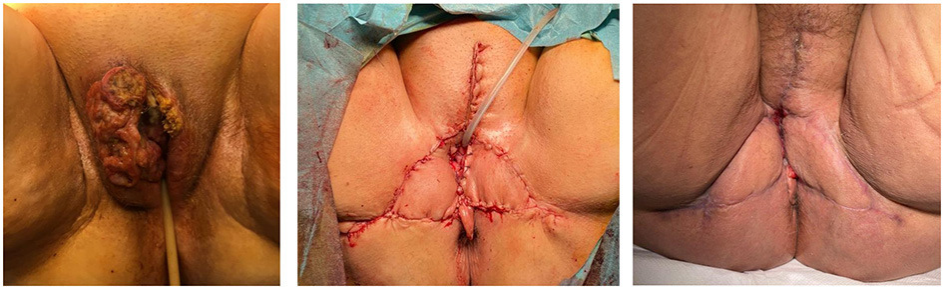
Bilateral fasciocutaneous advancement V-Y flap (preoperative, postoperative, and three-month follow-up).

**Figure 3:**
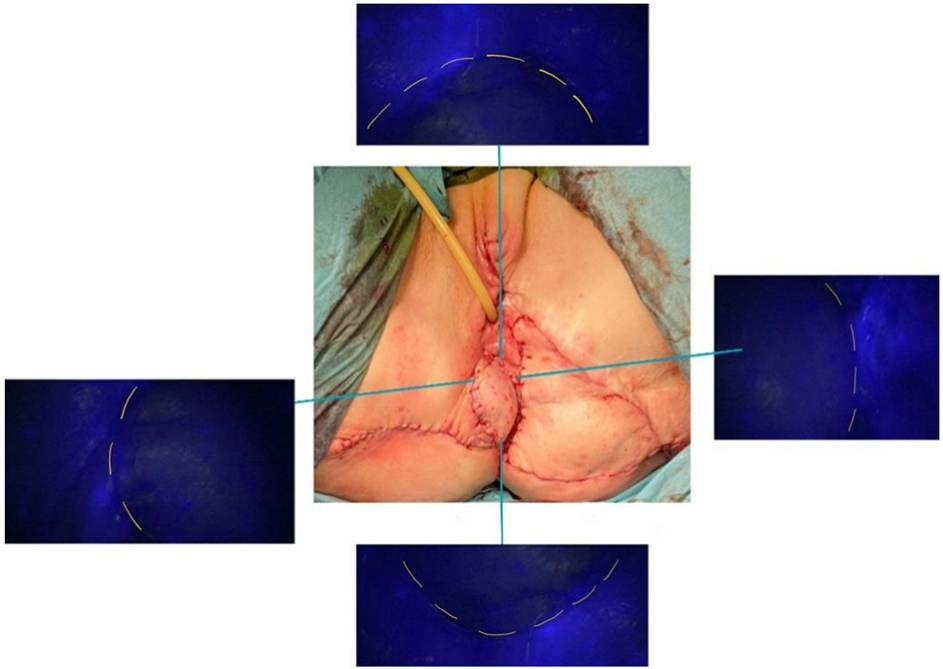
Combined V-Y and lotus petal flap with intraoperative dye uptake.

Figures 1–3 show the preoperative, intraoperative, and 3-month follow-up cases.

## Discussion

The present case series has confirmed the feasibility of the intraoperative technique of vulvar flap vitality assessment using indocyanine green and laparoscopic probe. No intra and postoperative procedure-related complications were reported.

In 1980, Magrina et al. first described how intravenous fluorescein with ultraviolet light allowed a good identification of poorly vascularized areas following radical vulvectomy (20). However, at the time, a single skin incision was performed for inguinal lymphadenectomy and radical vulvectomy significantly increasing the rate of postoperative complications compared to the double inguinal-vulvar incisions (2). Over the years, different methods have been described for flap vitality evaluation both in gynecologic and plastic surgery. Oliver et al. (21) with orthogonal polarization imaging, Furukawa et al. (22) with indocyanine-green fluorescence angiography, and Zhu et al. (23) *via* diffuse reflectance spectroscopy reported the reproducibility of different techniques to assess skin flap vascularity. Unfortunately, all of these methods require expensive machinery, often missing in common gynecological clinics.

To overcome this problem, we experimented with an evaluation of the vulvar flap viability using devices normally used in gynecological surgery for sentinel lymph node identification in endometrial, cervical, or ovarian cancer (24–29). Through the laparoscopic infrared probe and ICG dye was possible to evaluate the vascularization of the vulvar flap surgical margins. The dye administered intravenously will reach the margins of the surgical wound showing, where present, a good vascularization of the flap. In non-uptake of the dye cases, the flap is probably not adequately vascularized and could result in postoperative surgical dehiscence. Furthermore, in these cases, a resection of the surgical margins or the packaging of a new vulvar flap may be necessary.

We know that this method has limitations. The major limit is the subjective assessment of tracer uptake. Unlike angiography which shows an objective assessment of the degree of flap vascularization, with our method, the evaluation should be limited to the uptake vs. non-uptake. However, in line with our method and as reported by several authors, a standardized method for vulvar flap viability evaluation is still absent, and subjective clinical evaluation still represents one of the best methods for assessing vulvar flap vitality (30–32). Furthermore, it would be useful to prospectively evaluate the flap viability using ICG and infrared probes with only clinical evaluation to assess whether this new method allows better surgical outcomes. Furthermore, both the complication and the reoperation that could be avoided with this method would certainly improve the patients' quality of life, as reported for other surgeries (33–37).

To conclude, recent studies are successfully investigating the use of the combined ICG and technetium 99 m Nanocolloid for sentinel lymph node identification in vulvar cancer patients (38, 39). In line with these authors, we believe that the ICG is a safe method, in which the oncologist gynecologist is confident, and that could shortly find different uses in vulvar cancer patients. To date, a prospective multicenter study with a control group is planned to confirm the results of the present pilot study.

## Conclusions

Vulvar flap viability assessment using Indocyanine green and a laparoscopic infrared probe is a feasible method. All cases included in the analysis showed a dye uptake on the surgical edge of the flap. Further, prospective studies are needed to confirm the method in clinical practice and to evaluate its superiority over simple subjective clinical evaluation.

## Data Availability Statement

The raw data supporting the conclusions of this article will be made available by the authors, without undue reservation.

## Ethics Statement

The studies involving human participants were reviewed and approved by University of Parma. The patients/participants provided their written informed consent to participate in this study.

## Author Contributions

VC and RB: project development, data collection, manuscript writing, and editing. LM, GA, GS, and AR: data collection and manuscript writing. SG, FC, AE, and SC: protocol/project development and manuscript writing. All authors contributed to the article and approved the submitted version.

## Conflict of Interest

The authors declare that the research was conducted in the absence of any commercial or financial relationships that could be construed as a potential conflict of interest. The reviewer [ET] declared a shared affiliation, with no collaboration, with the authors [AR and SG] to the handling editor.

## Publisher's Note

All claims expressed in this article are solely those of the authors and do not necessarily represent those of their affiliated organizations, or those of the publisher, the editors and the reviewers. Any product that may be evaluated in this article, or claim that may be made by its manufacturer, is not guaranteed or endorsed by the publisher.
